# Integration of a Bimanual Training Program Using Joystick-Operated Ride-On Toys into an Intensive, Task-Oriented Hybrid Intervention for Children with Unilateral Cerebral Palsy: A Feasibility Study

**DOI:** 10.3390/jcm14248672

**Published:** 2025-12-07

**Authors:** Kush Kataria, Patrick D. Kumavor, Sudha Srinivasan

**Affiliations:** 1 Physical Therapy Program, Department of Kinesiology, University of Connecticut, Storrs, CT 06269, USA; kkataria@uchc.edu; 2 Biomedical Engineering Department, University of Connecticut, Storrs, CT 06269, USA; patrick.d.kumavor@uconn.edu; 3 Institute for Health, Intervention, and Policy (InCHIP), University of Connecticut, Storrs, CT 06269, USA; 4 Institute for the Brain and Cognitive Sciences (IBACS), University of Connecticut, Storrs, CT 06269, USA

**Keywords:** ride-on toys, bimanual training, unilateral cerebral palsy, novel intervention, clinician-delivered training, technology-based therapy adjunct, hemiplegia, upper extremity

## Abstract

**Background/Objectives**: We studied the feasibility of incorporating a play-based bimanual ride-on-toy navigation training (RNT) program into an intensive hybrid training camp based on principles of modified constraint induced movement therapy and bimanual training for children with unilateral cerebral palsy (UCP). The bimanual RNT sessions included theme-based play involving navigational exploration and object-based tasks. **Methods**: We employed a pretest-posttest, mixed methods design. Ten children between 3 and 11 years participated. Camp was 6 h/day and 5 days/week for 3 weeks. Researchers trained camp staff to provide RNT every day. The feasibility of clinician delivery of RNT was assessed using training logs and staff exit questionnaires. The combined effects of the camp programming, inclusive of bimanual RNT, was measured through a combination of standardized tests (Quality of Upper Extremity Skills Test (QUEST), Shriner’s Hospital Upper Extremity Evaluation (SHUEE), and Box and Blocks Test) and video-coding measures. We report on means (M), standard errors (SE), and effect sizes (ES) with 95% confidence intervals for outcome measures. **Results**: The average session adherence was 90.7%, and staff were able to successfully deliver RNT, despite initial logistical challenges. In combination with camp programming, RNT led to improvements in the total QUEST score (pretest M (SE): 77.54 (5.11), posttest M (SE): 81.46 (5.22)) and SHUEE spontaneous functional analysis score (pretest M (SE): 41.33 (7.48), posttest M (SE): 50.22 (7.88)). Children increased the use of their affected upper extremity (UE) during late RNT sessions and improved in their navigational abilities. **Conclusions**: RNT is a fun and easily adaptable therapy adjunct that can complement traditional therapies to incentivize spontaneous use of the affected UE in children with UCP.

## 1. Introduction

Cerebral palsy (CP) is a neurological condition diagnosed in infancy or early childhood, where atypicalities in brain development result in motor impairments [1]. Cerebral palsy can manifest in various ways, with over 35% of children with CP having unilateral cerebral palsy (UCP), meaning one side of their body is impaired in terms of function and mobility [2,3], with the upper extremity (UE) usually more affected than the lower extremity [2,4]. This makes it difficult for children with UCP to independently complete tasks of daily living and partake in play and recreational activities, potentially impacting their overall quality of life [2,5]. Evidence-based practice guidelines recommend interventions that involve intensive, repetitive, and task-oriented practice involving the affected UE to improve motor function in children with UCP [2,6]. Modified constraint-induced movement therapy (mCIMT) and bimanual training (BT) are the two most recommended evidence-based UE interventions [7,8]. While mCIMT involves restraining the child’s unimpaired UE for several hours (~6 h) per day while completing training activities with the affected arm, BT focuses on two-handed, coordinated motor practice [7,8].

Though both mCIMT and BT approaches are very effective in improving UE function in children with UCP, it may be challenging to sustain child motivation during such intensive training programs [7,9,10]. Different groups have tried to increase the child friendliness of these interventions by using themed activities or incorporating novel and immersive technologies into conventional interventions [11,12,13,14,15,16,17,18,19]. For example, Miller and colleagues incorporated circus-themed UE training activities (i.e., putting on circus costumes, practicing juggling, etc.) for 2 h/day as part of a 10-day hybrid mCIMT + BT camp and found that it led to greater child engagement when compared to the control group that received standard occupational therapy [11]. The authors highlight that the novelty and fun of participating in a circus-themed day camp led children to perceive these intensive tasks as exciting challenges while reducing frustration [11]. Similarly, Green et al. investigated the effects of incorporating magic-themed activities into hand-arm bimanual intensive training (HABIT) offered as 2-week intensive summer camp programs in Israel and the UK for children with hemiplegia. The program was developed in collaboration with professional magicians and included preparatory activities such as making props and costumes for a final magic show as well as the practice of bimanual magic hand tricks. These tricks gradually increased in complexity, timing, accuracy, and fine manual dexterity across the duration of the program. They found that children showed significant improvements in the quality of affected UE use during bimanual tasks on the Assisting Hand Assessment, functional independence and affected UE use during daily activities measured using the Children’s Hand Experience Questionnaire, and time to perform functional tasks on the Jebsen Taylor Test of Hand Function [12].

In the realm of using technology to aid rehabilitation for children with UCP, researchers have explored innovative interventions such as virtual reality, robotics, Nintendo Wii, and exergames to enhance child motivation and motor function [13,14,15,16,17,18,19,20,21]. For example, Gilliaux and colleagues examined whether adding robot-assisted therapy (using a distal effector robot REAPlan) to conventional physical and occupational therapy improved upper-extremity function in children with UCP. In their single-blind randomized controlled trial with 16 children, the experimental group received three sessions of conventional therapy + two sessions of robot-assisted therapy per week for 8 weeks, whereas the control group received five sessions/week of conventional therapy for 8 weeks. They found that the experimental group showed greater improvements in movement smoothness and manual dexterity compared to the control group [14]. Similarly, Roberts and colleagues delivered an exoskeleton-based virtual reality gaming intervention for 30 min/day to improve affected UE function in 31 children with UCP as part of a 2-week, 60 h pirate-themed mCIMT summer camp [19]. They found that the technology adjunct was well-received, feasible to implement, and the overall program led to clinically significant improvements in bimanual skills and occupational performance [19]. Despite the benefits associated with the use of technology-based adjuncts for UE rehabilitation, there are challenges associated with their integration into clinical practice, such as the equipment being bulky, need for interventionists to receive specialized training to operate the technology, high cost of the technology, and some technologies not being easily customizable to children’s needs/goals [20,21]. Therefore, there is a need for more accessible, versatile, and adaptable technology-based UE interventions for children with UCP that provide clinicians with engaging and effective tools to boost child motivation and therapeutic dosing.

To address some of these gaps identified in past research, our group has been exploring the use of training programs incorporating joystick-operated ride-on toys as an engaging, technology-based, and child-friendly therapy adjunct that can incentivize use of the affected UE in children with hemiplegia. Over the course of multiple pilot studies, we have developed a comprehensive, manualized ride-on-toy navigation training (RNT) program that combines navigational training opportunities with themed object-based UE tasks [22,23,24,25,26]. The RNT program involves the use of modified commercially available joystick-operated ride-on toys to encourage children to use their affected upper extremity in purposeful ways that support task-focused and repeated motor practice. Specifically, children are encouraged to use both arms in a coordinated fashion to control the joysticks and move the toy through navigational courses set up in their physical environment. They also complete tasks involving objects positioned along the route, which promotes the practice of functional UE movement patterns that are typically used during daily activities. The RNT program is based on the principles of self-determination theory of motivation and motor learning theories [25,27,28,29]. The program is aimed to promote child autonomy (providing children with choices, using child-preferred themes), relatedness (choosing age-appropriate activities that would increase children’s sense of connection with peers and siblings), and competence (ensuring that children experience a sense of success and confidence through choice of activities at the optimal challenge point), all of which foster their motivation to engage in and persist with the program. Similarly, we use principles of motor learning including promoting variable practice, creating a just-right challenge for each child, providing multimodal feedback and reinforcement, and using a least-to-most prompting hierarchy [24,25,26].

This paper reports on the feasibility of training clinicians to deliver this intervention as part of a hybrid mCIMT + BT summer camp with 10 children with UCP between 3 and 11 years of age. The goal of this line of research is to develop a suite of engaging, technology-based training tools that clinicians can incorporate to boost and sustain child motivation during intensive, task-oriented training programs. Previously, we reported that the supplemental RNT program was well-received by children and clinicians and led to high levels of child engagement throughout the duration of the camp [29]. In this paper, we describe the nature of opportunities afforded by RNT for affected arm use and bimanual motor practice. We also report on the combined effects of the overall camp programming, inclusive of RNT, on affected UE motor function in children with UCP, assessed using a combination of standardized tests and training-specific behavioral measures. We hypothesized that the bimanual RNT intervention would be successfully implemented by camp staff and, when combined with other camp activities, would lead to improvements in the children’s unimanual and bimanual motor abilities. In the sections that follow, we describe the study methods, including participants, camp structure, and the RNT program; present results on feasibility and upper extremity outcomes; discuss these findings in relation to the existing rehabilitation literature; and conclude with the study’s limitations and implications for future research and clinical practice.

## 2. Materials and Methods

### 2.1. Participants

We employed a single-group, pretest-posttest, mixed methods study design. Ten children with UCP (mean age (SD): 6.99 (2.13) years; 4M, 5F; 5 left-sided hemiplegia, 5 right-sided hemiplegia) who participated in an annually held summer camp in Western Connecticut for children with UCP volunteered to participate in our study. Children with clear asymmetry in UE strength/control were included. Exclusion criteria included children with a recent history (within the last 6 months) of UE trauma/surgery, unable to sustain supported sitting for 20 min, with blindness/profound visual impairment, unable to activate the joystick using their affected UE, unable to follow 2-step verbal instructions, or those who exceeded the weight limits of the toy (>150 lbs.). Participating children had low to moderate severity of UE impairment as classified using the Manual Ability Classification System, a tool that assesses how children with CP use their hands when handling objects of daily activities, where higher scores indicate greater levels of impairment and lower manual ability [30]. Three children had a MACS score of I, five children had a score of II, and two children had a score of III. Furthermore, two children had additional comorbidities of epilepsy and/or tuberous sclerosis [29]. Six clinicians/camp staff (all females, >18 years; 1 pediatric physical therapist (PT) who was the camp director, 2 occupational therapists (OT), and 3 occupational therapy graduate students) also participated in the study. These clinicians were involved in co-designing the RNT program with the research team, delivering RNT with children, and sharing their perspectives on the feasibility of RNT at the end of the program. The study was approved by the Institutional Review Board at the University of Connecticut. We obtained parental permission and child oral or written assent prior to the study. Camp staff were provided with an information sheet describing study procedures.

### 2.2. Setting and Camp Structure

The camp was designed as a 3-week, 5-days/week, intensive full-day (9 am–3 pm) program for children with UCP. It followed a hybrid training format, combining principles of mCIMT and BT (i.e., 5 h/day of unimanual affected UE training while constraining the less-affected arm, and 1 h/day of bimanual practice without constraining the less-affected arm) [31,32,33,34]. In 2022, our research team provided the bimanual RNT program to children with UCP as part of this annual camp training. We found high levels of child engagement during RNT activities and children wanted to participate in the program again. Both clinicians and caregivers felt that RNT was a valuable addition to existing camp programming, specifically, in promoting child engagement and intrinsic motivation, serving as a reward during the camp day, facilitating targeted practice of bimanual coordination skills, and increasing spontaneous use of the affected arm [24,25,26]. Based on the positive findings of our previous study, the camp director and staff reached out to our research team and indicated interest in incorporating RNT again into the camp in 2024. Specifically, they were interested in building capacity among camp staff members in delivering the RNT program with children following appropriate training from the research staff. Therefore, in view of the aims of the present study, camp staff were closely involved from the outset in co-developing the study protocol with the researchers in the 3–4 months leading up to the start of the camp. Different days at camp were structured around different themes (e.g., career day, baking, sports, animated movies, etc.), and the RNT session activities for that day were aligned with the camp theme. The research team met with the camp staff for a 2 h in-person training session one week prior to the start of camp to review study goals and procedures and distribute printed RNT materials (detailed manuals with session specific instructions, session-themed props, and ride-on toy device user manual). Thereafter, during the duration of the actual 3-week camp, researchers visited the camp site 1–2 times/week to videotape RNT sessions, support camp staff-delivery of RNT (as needed), and trouble-shoot any device-related issues (if applicable).

### 2.3. Ride-on-Toy Navigation Training (RNT) Program

**Figure 1:**
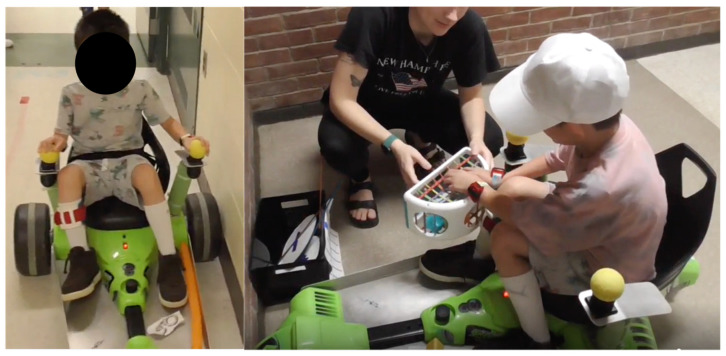
Example of a child completing RNT activities of navigation and object-based UE tasks.

We modified the commercially available, dual joystick-operated ride-on toy called the Huffy Green Machine Vortex (Huffy Corporation, Dayton, OH, USA) by raising the joystick height, adding aluminum forearm and leg support plates, and attaching foam balls on the joysticks for easier grasp (See Figure 1). To move the toy forward, children had to push both joysticks forward simultaneously, to move backward they had to pull back on both joysticks simultaneously, to move to the right they had to push with the left joystick and pull back with the right, and to move to the left they had to push with the right and pull back with the left arm. The RNT program consisted of 14 sessions delivered by trained camp staff (PTs, OTs, and OT students) over the 3 weeks of camp. Each session consisted of 2 parts: 1. Navigating skillfully through multiple obstacle courses involving increasingly complex paths, such as straight paths, arcs, and slalom courses. 2. Object-based bimanual and unimanual UE tasks that the children completed at intermediate checkpoints during navigation while still seated in the toy (see Figure 1). These themed tasks included both fine and gross motor activities (e.g., reaching, lifting, grasping, releasing, opening, throwing, catching, knocking, etc.), and clinicians were encouraged and provided ideas to tailor the activities to each child’s specific abilities. Exemplar session activities for a baking-themed session involved the child navigating a slalom path and completing tasks along the way to collect their baking supplies and utensils (e.g., opening a jar to get the dessert recipe, lifting cones to find their apron and chef hat hidden underneath, and knocking down cups to find ingredients such as flour and eggs). The key ingredients and session activities of the RNT program were developed, tested, and modified based on stakeholder feedback (from children, families, and clinicians) over the past 4 years through a series of pilot studies [22,23,24,25,26].

### 2.4. Outcomes and Measures

We assessed the feasibility of clinician delivery of RNT by tracking adherence using training logs and asking clinicians at the end of camp about their impressions related to RNT delivery using exit questionnaires. In this study, we report on adherence and clinician perceptions on the ease of RNT delivery. Details of all other data collected from clinician exit questionnaires related to acceptability and perceived impact of RNT are reported in a different publication [29,35]. To assess the combined impact of the task-oriented camp programming, inclusive of RNT, we used a combination of standardized tests (administered before and after the camp) and training-specific measures (assessed using early and late RNT session videos). For all standardized and training-specific measures, two coders coded 20% of the total videos to establish an inter- and intra-rater reliability of over 90% [22,24,25,26]. All discrepancies between raters were resolved through consensus coding and discussion with the first and last authors.

#### 2.4.1. Adherence and Clinician Impressions on Delivery of RNT

We kept track of the number of sessions completed and the average session duration for each child using staff-reported training logs. Clinicians were also asked about their impressions related to feasibility/ease of RNT delivery at the end of the camp using 5-point Likert-style questions, where higher ratings corresponded to higher levels of feasibility and ease of RNT delivery.

#### 2.4.2. Testing Measures Conducted at Pretest and Posttest

##### Shriner’s Hospital Upper Extremity Evaluation (SHUEE)

The Shriner’s Hospital Upper Extremity Evaluation (SHUEE) is a validated, video-based test comprising 16 tasks that measure bimanual motor function in children with UCP ages 6–15 years [36]. The test evaluates how well children spontaneously use their affected UE in nine of sixteen tasks, known as spontaneous functional analysis (SFA). The SFA is rated on a 6-point scale, from no use of the affected UE (score 0) to spontaneous and optimal use (score 5). Additionally, all 16 tasks are assessed for dynamic segmental alignment of the affected UE at the elbow, forearm, wrist (sagittal and frontal planes), thumb, and fingers, referred to as dynamic positional analysis (DPA). This is scored on a 4-point scale, ranging from pathological (score 0) to typical alignment (score 3) [36]. The maximum possible SPA score is 45, and the maximum possible DPA score is 72; we report on changes in percent total SFA and DPA scores from pretest to posttest (i.e., from before to after the 3-week camp-based intervention).

##### Quality of Upper Extremity Skills Test (QUEST)

The Quality of Upper Extremity Skills Test (QUEST) is a criterion-referenced, reliable, and validated test that assesses UE motor function in children with CP ages 2–16 years [37,38]. The test includes 36 unimanual items scored on a dichotomous scale in 4 domains: dissociated movements, grasps, protective extension, and weight bearing. Scores for each sub-domain and the total score are calculated as percentages. The total QUEST score is calculated by summing scores for each sub-domain test divided by the total number of sub-domains tested. The total scores on the QUEST range from 0 to 100. Higher scores indicate better UE movement quality and range of motion [37,38].

##### Box and Blocks (BBT)

This timed test involves a box with 150 blocks (each block of size 2.54 cm × 2.54 cm × 2.54 cm) and children are asked to move blocks, one at a time, as quickly as they can across a central partition using one hand. Children completed the 1 min task with their affected and unaffected hands, and we report on the number of blocks successfully transported within the minute by each hand [39]. The BBT is a reliable and validated assessment for children with UCP ages 5–12 years [40].

##### Path Navigation Test

This custom designed test evaluates children’s ability to successfully use both hands to drive the ride-on toy through a straight, a circular, and a slalom path. We report on the frequency of navigational errors (i.e., child bumps against fixed objects, such as walls or doors, or moveable objects, such as cups or cones used to demarcate the path, or child drives outside the demarcated path) from the pretest to posttest.

#### 2.4.3. Training-Specific Measures Analyzed from Early to Late Sessions

For these measures, videos of one early (week 1) and one late (week 3) training session per child were coded on a millisecond-by-millisecond basis using the Datavyu video coding software (version 1.5.3) [22,26]. The following behaviors were coded during early and late RNT sessions.

##### Affected Arm Use During Training Session

Early and late training sessions were coded to assess the extent of unimanual and bimanual arm use during navigation and the intermediate, object-based tasks in each RNT session. Specifically, we classified arm use during navigation and tasks as either being appropriate or inappropriate. Appropriate for navigation referred to bimanual arm use to drive the dual-joystick operated ride-on toy and inappropriate referred to unimanual arm use. For tasks, appropriate arm use was defined as instances when the child used their affected arm during unimanual or bimanual tasks, and inappropriate when they solely used their unaffected arm to complete the activity. We report on the percent of navigation time and the percent of task time spent in appropriate UE use during early and late sessions.

##### Level of Independence During Ride-On Toy Navigation

The use of ride-on toys, specifically, purposeful arm-controlled navigational activities to promote UE motor function among children with UCP, is highly innovative. Children with UCP have difficulties in bimanual coordination skills [2]. Given the novelty of the navigational component of RNT, and the associated underlying requirement for coordinated bimanual skills to maneuver the toy, we also assessed children’s level of independence during navigation. Bouts of navigation (i.e., ride-on toy was in motion) during early and late training sessions were classified as being independent (i.e., child able to use affected arm independently without adult assistance to control the joystick), partially assisted (i.e., child requires some assistance from the adult to use their affected arm, for example, adult may hold the child’s hand in contact with the joystick or may stabilize the child’s elbow, but the child is still able to actively exert force to push/pull the joystick), or totally assisted (i.e., child is unable to use their affected arm actively and the adult maneuvers the joystick for the child). Note, children did not require any assistance in using their unaffected arm to control the joystick on their unaffected side. We report on the percent of navigational session time spent in independent ride-on toy navigation during early and late sessions.

### 2.5. Statistical Analysis

Our feasibility study was not powered to detect significant intervention effects. Instead, our preliminary analyses are meant to obtain estimates of means (M), standard errors (SE), effect sizes (ES), and 95% confidence intervals (CI) around the effect sizes to inform power calculations for our future planned larger trial after adjusting for the sample size of this study. Given our small sample, we will report both group-level descriptive statistics and individual-level data on the number of children who followed the group trends. We report the ES (Cohen’s d with a Hedges’ g correction for small sample sizes) for measures where the 95% CI around the ES does not include zero (suggestive of a non-zero intervention effect). We interpret ES using Cohen’s conventions of small (0.2–0.49), medium (0.5–0.79), or large (0.8 and above) [29].

## 3. Results

### 3.1. Adherence

The average session adherence was 90.7%, with four out of ten children having 100% attendance. One child missed five RNT sessions (due to a planned vacation), another child missed two sessions (due to sickness), and four children missed one session (due to sickness or technical difficulties, i.e., toy not charged properly). All camp staff reported being able to deliver RNT all days of the week. None of the participants refused to engage in RNT activities during the 3-week program. Three out of six staff (50%) rated RNT as being “very” feasible to deliver, and the remaining three (50%) rated it as “somewhat” feasible to deliver. Primary challenges identified were related to staffing shortages, logistics of scheduling RNT for all 10 children within the camp day and managing RNT session delivery along with other camp activities. To address these identified challenges, camp staff suggested that they would designate one lead staff member along with one student helper to manage and oversee RNT sessions in future iterations of the camp.

### 3.2. Testing Measures Conducted at Pretest and Posttest

#### 3.2.1. Shriner’s Hospital Upper Extremity Evaluation (SHUEE)

Children showed improvements in total SFA scores from pretest to posttest (pretest M (SE): 41.33 (7.48), posttest M (SE): 50.22 (7.88), ES (95%CI): 0.88 (0.17–1.56), with 9 out of 10 children following the group trend, see Table 1 for individual data). Changes in DPA scores were more non-uniform across children.

#### 3.2.2. Quality of Upper Extremity Skills Test (QUEST)

Children showed improvements in the total QUEST scores from pretest to posttest (pretest M (SE): 77.54 (5.11), posttest M (SE): 81.46 (5.22), ES (95%CI): 1.29 (0.50–2.29)), specifically, in dissociated movements (pretest M (SE): 77.03 (5.07), posttest M (SE): 81.25 (4.36), ES (95%CI): 0.81 (0.12–1.47)) and grasps (pretest M (SE): 72.59 (5.27), posttest M (SE): 77.04 (6.09), ES (95%CI): 1.06 (0.30–1.79)) subdomains (see Table 2). All 10 children improved in total scores of the QUEST, 9 children improved on the dissociated movements sub-domain, and 7 children improved in their grasping abilities from pretest to posttest (see Table 2 for individual data).

#### 3.2.3. Box and Blocks (BBT)

Children demonstrated asymmetry between the UEs with greater blocks being transferred in 1 min using the unaffected compared to the affected arm across both testing sessions (affected UE M (SE): 13.00 (1.54), unaffected UE M (SE): 28.89 (1.96), ES (95%CI): 1.98 (1.18–2.76)). There were no notable training-related improvements in performance on the Box and Blocks Test from pretest to posttest (affected UE M (SE)—pretest: 12.67 (2.19), posttest: 13.33 (2.29); unaffected UE M (SE)—pretest: 28.78 (2.64), posttest: 29.00 (3.06)).

#### 3.2.4. Path Navigation Test

There was a decrease in the total number of navigational errors while traveling through the test paths from the pretest to posttest (pretest M (SE): 40.4 (9.09), posttest M (SE): 19.6 (4.26), ES (95%CI): 0.98 (0.24–1.68)), with all 10 children showing a decrease in navigational errors.

### 3.3. Training-Specific Measures Analyzed from Early to Late Sessions

#### 3.3.1. Affected Arm Use During Training Sessions

On an average, children showed high levels of task-appropriate bimanual UE use for the navigational portion of training sessions, with bimanual use staying consistently high across sessions for most children (see Table 3). Arm use during object-based tasks was more variable, and children showed improvements in task-appropriate UE use from early to late sessions (early M (SE): 54.91 (9.12), late M (SE): 70.41 (6.41), ES (95%CI): 1.06 (0.31–1.79), with 9 out of 10 children followed this group trend, see Table 3).

#### 3.3.2. Level of Independence During Ride-On Toy Navigation

There was an increase in the percent of navigational session time spent in independent ride-on toy navigation from early to late training sessions (early M (SE): 85.36 (5.61), late M (SE): 98.37 (0.92), ES (95%CI): 0.73 (0.06–1.4), with all 10 children showing improvements, see Table 3).

## 4. Discussion

Our study suggested that the RNT program was feasible for clinicians to deliver within an intensive task-oriented hybrid UE training program for children with UCP. Some challenges associated with RNT delivery were related to staffing and scheduling. Staff members provided clear suggestions to address these issues for future iterations of RNT integration into camp programming. Moreover, in combination with all other camp activities, RNT led to improvements in the children’s bimanual and unimanual motor function as assessed using standardized motor tests. Across training weeks, children also increased the use of their affected UE during RNT sessions and improved their navigational skill and accuracy.

Our past research has demonstrated the feasibility of delivery of RNT by trained research staff [22,26]. In these studies, we also demonstrated that the RNT program, in combination with mCIMT + BT, led to improvements in spontaneous affected UE use and motor capacity assessed using the QUEST and SHUEE [23,24,25,41]. The present study replicated our past findings and further extended it to a real-world context (i.e., having clinicians incorporate RNT as part of an intensive, task-oriented UE training camp). Despite the wide confidence intervals around our effect size estimates for the standardized tests owing to the small sample size in our study, it is meaningful to see that most of the children showed improvements in performance after only 3 weeks of intensive hybrid UE therapy, inclusive of RNT. Furthermore, it is very promising to see the positive trends carry over when camp staff delivered the RNT program, and it speaks to the easy-to-implement and versatile nature of RNT. Following a 2 h in-person training and ongoing support from researchers as needed, camp staff were able to learn the principles of RNT and deliver the intervention with similar intensity as the research team. Though not reported here, we also observed an increase in the children’s scores on the clinician-filled ABILHAND-Kids Questionnaire [42], which assesses the child’s ability to complete 21 bimanual tasks of daily living [29,42].

Our findings are in line with other studies that have used technology-based adjuncts to supplement conventional therapy models aimed at improving UE motor function among children with UCP [13,14,15,16,17,18,19]. For example, in a retrospective analysis of their group-based Personalized Upper Limb Intensive Therapy (PULIT) program, which consisted of 30 h of therapy (combining mCIMT, BIT, and exergame-based robotics) delivered over 8 days, Bono and colleagues observed significant improvements in children’s manual dexterity and reported achievement of individualized goals related to daily activities within a short period of time compared to longer-duration conventional therapies. Like our study, they hypothesized that the high dose of practice within a motivating therapy environment may have contributed to these accelerated gains [13]. We found that the RNT program was well-received by children and clinicians and was perceived as a beneficial addition to existing camp programming to boost affected UE use. Our findings are also aligned with Roberts et al. who examined the acceptability associated with incorporating an upper extremity exoskeleton and virtual reality-based games through the Hocomo Armeo^®^ Spring Pediatric device as part of a 2-week mCIMT camp. Children received training with the Armeo^®^ device 30 min per day. They found that the training was feasible to implement within the camp context, was well-accepted by children, and when combined with camp programing led to improved unimanual and bimanual skills [19]. In contrast with some technologies that use bulky equipment, are costly, require extensive training, and are limited for use within lab settings, our RNT program uses easy-to-operate ride-on toys, is adaptable according to child needs, and can be easily set up across a variety of indoor and outdoor settings, that potentially expands the versatility and applicability of the program for children and families with diverse needs.

It should be noted that we did not see robust improvements in movement timing (Box and Blocks Test), gross motor skills (weight bearing, and protective extension domains of the QUEST), and dynamic alignment of UE joints during bimanual activities (DPA scores on the SHUEE). These findings may be explained by the principles of motor learning that emphasize the task-specific nature of gains following rehabilitation interventions [28]. Specifically, children showed improvements in skills that were directly related to the types of activities practiced during training sessions. Camp staff encouraged children to use their affected UE without emphasizing the speed of movements (i.e., children were not told to go as fast as possible, rather they were asked to take their time and focus on task performance/completion) or without providing a set prescriptive way of completing the task (i.e., independent completion of the task was emphasized rather than focusing on positions of individual joints during the activities), which may explain our findings.

Our findings add to the literature demonstrating the feasibility of training therapists, caregivers, and other stakeholders to deliver technology-based interventions with children with hemiplegia [43,44,45]. For example, Phelan and colleagues [15] explored the feasibility of an immersive virtual reality system to improve upper limb motor function in eight children with upper limb motor impairment ages 7–16 years. The researchers trained an OT on operation of the VR system, and the OT in turn helped with the first session and trained the caregivers on device operation for future sessions. The researchers found that the VR system was easy to learn and feasible, and all children improved in their affected limb’s range of motion, especially at the wrist and elbow. The researchers attributed these gains in UE function to the nature of the VR training, specifically the alignment of VR games with the types of skills practiced in conventional rehabilitation [15]. Along these lines, our manualized RNT program, inclusive of navigational and object-based activities, involved practice of a range of functional gross and fine motor movement patterns (e.g., reaching, pulling, pushing, lifting, grasping, pinching, opening, closing, squeezing, catching, throwing, holding, knocking, and twisting motions) using a variety of props/supplies (e.g., cups, cones, bean bags, jars, balls, etc.) in novel and playful ways. Therefore, this repeated, task-focused practice when combined with other camp programming, may have contributed to improvements in children’s UE motor skills.

The RNT program was designed to foster children’s motivation and participation in camp programming. Motivation is crucial in improving motor abilities in children with CP [46,47] and a lack of motivation may prevent children from partaking in intensive, repetitive, and task-oriented UE motor practice that current research guidelines recommend for children with UCP [2,6]. In this context, the feedback from our previous pilot studies shows that children do not perceive RNT as “therapy” and think of it as a playful game instead [22,26]. Ride-on toys are fun for children with and without disabilities, and when coupled with themed and purposeful object-based games afford multiple and diverse opportunities for children with UCP to use their affected UE in a sustained manner over the course of the RNT session. In line with our program/session design, several other groups have also used themed activities (e.g., pirate camp, circus-themed activities, magic camp) to increase child engagement and enjoyment during training sessions [11,12,19]. We found that the use of child-friendly themes increased children’s excitement to participate in training activities, and we were able to encourage children to practice functional movement patterns within the context of the session theme (e.g., practice opening a jar to “find Moana’s friends”). Similarly, in their circus-themed hybrid CIMT camp, Miller and colleagues mentioned practicing functional skills like dressing and ball manipulation in the context of children “trying on” their circus costumes or practicing juggling skills in preparation of a final circus concert [11].

As the RNT program progressed, children used their affected UE spontaneously and independently during tasks and navigation, which may have contributed to improvements in motor function through practice-based learning. Motor learning principles recommend the use of progressively challenging activities that provide a “just-right” challenge and foster exploration [28,48,49]. While camp staff were given ideas to make the activities harder and easier during the pre-camp training, they also had the freedom to adjust activities based on their clinical judgment to provide the appropriate challenge for each child. The RNT tasks did not require any technology or complex props, so they could be modified easily per each child’s ability (e.g., manually resisting child efforts while reaching for toys, using heavier balls for catch–throw activities, adding obstacles along the path to increase complexity of the navigational task, increasing speed of the toy for a greater challenge, etc.). The intrinsically motivating nature of RNT, the inherent flexibility available in the program for task customization per individual child needs, and the variable and distributed practice of functionally important movement patterns afforded during RNT sessions may be some of the key reasons why RNT, in combination with all other camp activities, contributed to children’s improved motor performance on the standardized tests in the posttest compared to the pretest.

Our work aligns with and adds to the findings of other innovative, playful, and novel interventions that aim to incentivize UE use and motor practice among children with UCP [13,14,15,16,17,18,19]. For example, Acar et al. [16] explored the use of Nintendo Wii games to supplement conventional therapy through a randomized control trial in 30 children with UCP ages 6–15 years. Trained physical therapists delivered the intervention twice a week for 6 weeks with each session lasting 45 min. Researchers found that the experimental group showed significant improvements in unimanual motor function on the QUEST [16]. Similarly, Peramalaiah et al. [17] used a randomized controlled trial design to test the feasibility and value of their robotic manipulandum device (RMD) that facilitated the practice of object manipulation skills through game-based exercises in 34 children with CP between 4 and 10 years of age. When compared to the control group, which received only conventional therapy, the experimental group showed greater improvements in grasping and manual dexterity skills [17]. Overall, we provide preliminary evidence supporting a play-based training program using ride-on toys that may be used to gamify conventional therapy, sustain child engagement during intensive interventions, and promote spontaneous affected UE use and motor function when incorporated into conventional UE rehabilitation interventions.

### Limitations

The generalizability of the findings from our pilot feasibility study is limited by the lack of a control group that only received camp programming without RNT sessions, a small heterogenous sample, brief study duration, and the absence of follow-up testing to assess carryover of improvements beyond the duration of the camp. Our findings are also limited to children with a mild-to-moderate level of manual impairment as assessed using the MACS (levels I–III). In the future, we would like to assess the effects of RNT for children with more severe impairments (i.e., MACS levels IV and V). Per clinician reports, although child interest and engagement were sustained over the 3-week duration, it remains to be assessed if children will continue to be motivated to practice RNT sessions beyond this duration. We recommended consistent staffing for RNT sessions with each child; however, due to scheduling constraints, other camp staff occasionally stepped in, which may have introduced variability in session quality across weeks.

## 5. Conclusions

This is the first pilot study to assess the incorporation of a bimanual, clinician-led UE training program using ride-on toys to boost child engagement, purposeful arm use, and bimanual motor practice within an intensive, hybrid UE training camp for children with UCP. Following a collaborative researcher and clinician co-led pre-camp planning process and a research staff-led training session, camp staff were able to successfully implement and incorporate RNT into the existing camp structure, with minimal challenges. Average adherence with RNT was 90.7% with all children complying with training activities. The combined task-oriented camp programming inclusive of RNT led to improvements in children’s affected UE spontaneous use and motor function, as assessed by standardized tests. Children showed increased use of their affected UE during later RNT sessions and improved their navigational skills. RNT is a fun, versatile, and easily adaptable therapy adjunct that clinicians can use to complement traditional therapies and promote affected UE motor function in children with CP. The findings from this study represent one way in which ride-on toys may be used as therapy adjuncts to foster child motivation and participation during intensive conventional rehabilitation programs. Future studies should assess the incorporation of RNT in other settings such as schools, hospitals, and out-patient clinics that provide care to children with UCP, using larger samples and more controlled designs.

## Figures and Tables

**Table 1:** Individual data on SHUEE scores at pretest and posttest.

Child Number		% Spontaneous Functional Analysis (SFA) Scores	% Dynamic Positional Analysis (DPA) Scores
1	Pretest	57.78	66.67
	Posttest	60.00	72.22
2	Pretest	55.56	68.06
	Posttest	66.67	73.61
3	Pretest	48.89	59.72
	Posttest	55.56	70.83
4	Pretest	0.00	55.56
	Posttest	6.67	50.00
5	Pretest	0.00	26.39
	Posttest	4.44	34.72
6	Pretest	57.78	76.39
	Posttest	53.33	68.06
7	Pretest	55.56	84.72
	Posttest	64.44	84.72
8	Pretest	26.67	41.67
	Posttest	51.11	69.44
9	Pretest	55.56	79.17
	Posttest	80.00	88.89
10	Pretest	55.56	88.89
	Posttest	60.00	88.89

**Table 2:** Individual data on QUEST total and subdomain scores at pretest and posttest.

Child Number		Total Score	Dissociated Movements	Grasps	Protective Extension	Weight Bearing
1	Pretest	73.87	70.31	40.74	94.44	90.00
	Posttest	81.28	84.38	40.74	100.00	100.00
2	Pretest	88.32	87.50	77.78	100.00	88.00
	Posttest	92.06	89.06	85.19	100.00	94.00
3	Pretest	69.37	59.38	77.78	58.33	82.00
	Posttest	71.05	64.06	81.48	66.67	72.00
4	Pretest	52.95	56.25	55.56	50.00	50.00
	Posttest	54.90	64.06	55.56	50.00	50.00
5	Pretest	56.40	62.50	55.56	55.56	52.00
	Posttest	59.57	64.06	55.56	66.67	52.00
6	Pretest	93.17	87.50	85.19	100.00	100.00
	Posttest	94.63	85.94	92.59	100.00	100.00
7	Pretest	95.91	98.44	85.19	100.00	100.00
	Posttest	97.22	100.00	88.89	100.00	100.00
8	Pretest	64.61	64.06	70.37	50.00	74.00
	Posttest	69.73	73.44	81.48	50.00	74.00
9	Pretest	87.33	93.75	88.89	66.67	100.00
	Posttest	96.98	95.31	92.59	100.00	100.00
10	Pretest	93.49	90.63	88.89	94.44	100.00
	Posttest	97.12	92.19	96.30	100.00	100.00

**Table 3:** Training-specific measures of affected UE use during early and late sessions.

Child Number		% Task-Appropriate UE Use: Navigation	% Task-Appropriate UE Use: Object Interactions	% Independent Navigation
1	Early	88.86	64.88	90.41
	Late	94.39	73.53	99.78
2	Early	89.95	65.22	97.22
	Late	87.07	75.96	99.88
3	Early	92.29	38.64	97.11
	Late	90.04	68.78	100.00
4	Early	70.08	6.78	88.90
	Late	68.09	24.14	99.04
5	Early	68.47	6.24	48.60
	Late	64.39	46.48	92.17
6	Early	93.34	72.26	96.89
	Late	96.74	83.93	100.00
7	Early	87.24	86.45	97.76
	Late	97.98	87.47	100.00
8	Early	78.17	53.86	88.72
	Late	75.68	82.14	93.68
9	Early	94.52	80.84	56.45
	Late	98.44	87.81	100.00
10	Early	91.28	73.99	91.55
	Late	90.67	73.74	99.16

## Data Availability

The datasets used and/or analyzed during the current study are available from the corresponding author on reasonable request.

## Abbreviations

The following abbreviations are used in this manuscript:

UCP	Unilateral Cerebral Palsy
RNT	Ride-on-toy Navigation Training
mCIMT	Modified Constraint-Induced Movement Therapy
BT	Bimanual Training
UE	Upper Extremity
IRB	Institutional Review Board
QUEST	Quality of Upper Extremity Skills Test
SHUEE	Shriner’s Hospital Upper Extremity Evaluation
DPA	Dynamic Positional Analysis
SFA	Spontaneous Functional Analysis
ES	Effect Size
SE	Standard Error
M	Mean
OT	Occupational Therapists
